# Post-Processing Effect on the Corrosion Resistance of Super Duplex Stainless Steel Produced by Laser Powder Bed Fusion

**DOI:** 10.3390/ma17122807

**Published:** 2024-06-08

**Authors:** Zbigniew Brytan, Mengistu Dagnaw, Jana Bidulská, Róbert Bidulský, Mohd Ridha Muhamad

**Affiliations:** 1 Department of Engineering Materials and Biomaterials, Faculty of Mechanical Engineering, Silesian University of Technology, 44-100 Gliwice, Poland; zbigniew.brytan@polsl.pl (Z.B.); mengistu.jemberu.dagnaw@polsl.pl (M.D.); 2 Department of Plastic Deformation and Simulation Processes, Institute of Materials and Quality Engineering, Faculty of Materials Metallurgy and Recycling, Technical University of Kosice, Park Komenského 11, 04001 Kosice, Slovakia; 3 Bodva Industry and Innovation Cluster, Budulov 174, 04501 Moldava nad Bodvou, Slovakia; director@biic.sk; 4 Advanced Research and Innovation Hub, Budulov 174, 04501 Moldava and Bodvou, Slovakia; 5 Centre of Advanced Manufacturing and Material Processing (AMMP Centre), Universiti Malaya, Kuala Lumpur 50603, Malaysia; ridha@um.edu.my

**Keywords:** LPBF, stress relieving, duplex stainless steel, corrosion resistance, EIS

## Abstract

This study examines the microstructural characteristics and corrosion resistance of super duplex stainless steel (SDSS) produced through laser powder bed fusion (LPBF). The analysis shows that the as-printed samples mainly exhibit a ferritic microstructure, which is due to the fast-cooling rates of the LPBF technique. X-ray and microstructure analyses reveal the presence of minor austenite phases in the ferritic matrix. The process of solution annealing led to a more balanced microstructure. Analyses of corrosion resistance, such as potentiodynamic polarization tests and EIS, indicate that heat treatment has a significant impact on the corrosion behavior of SDSS. Solution annealing and stress relieving at 400 °C for 1 h can improve corrosion resistance by increasing polarization resistance and favorable EIS parameters. However, stress relieving at 550 °C for 5 h may reduce the material’s corrosion resistance due to the formation of chromium nitride. Therefore, stress relieving at 400 °C for 1 h is a practical method to significantly enhance the corrosion resistance of LPBF-printed SDSS. This method offers a balance between microstructural integrity and material performance.

## 1. Introduction

Duplex stainless steels (DSSs) are a versatile class of steel with a two-phase microstructure consisting of approximately equal proportions of ferrite and austenite. They are known for their superior corrosion resistance and approximately twice the tensile strength of conventional austenitic stainless steels. The improved performance of these alloys, particularly in terms of resistance to pitting and stress corrosion cracking, is mainly due to their specific chemical composition, particularly the levels of chromium, molybdenum, and nitrogen. Super DSS alloys (SDSS), which have a higher alloy content than the DSS group, are designed for the most demanding corrosive environments [1,2].

**Figure 1:**
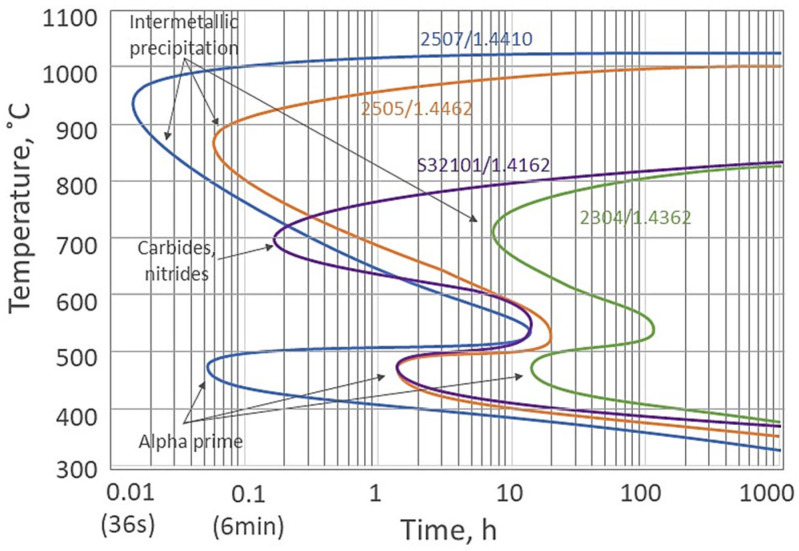
The isothermal precipitation diagram for various DSS is based on [4].

Duplex stainless steels are sensitive to intermetallic phases precipitations. Unwanted intermetallic phases, carbides, and nitrides can precipitate in these steels at various temperatures, particularly between 600 °C and 1000 °C. These include the sigma phase (σ), chi phase (χ), carbides (M_23_C6, M_7_C_3_), or nitrides (CrN, Cr_2_N), which can degrade their mechanical properties, particularly toughness, and reduce their corrosion resistance [3]. Furthermore, as the number of alloying elements (such as Cr, Mo, Ni) in DSSs increases, so too does the risk of secondary phase precipitation (as shown in Figure 1), which can affect both toughness and corrosion properties. This is particularly true for super DSS (SDSS) and high-alloy grades such as hyper duplex stainless steel (HDSS). To avoid secondary phase precipitation during heat treatment of DSS, the steel must be heated above 1000 °C and then rapidly cooled to dissolve any existing secondary phases and maintain a balanced microstructure [2,4]. It is essential to keep the steel outside the critical temperature range (as shown in Figure 1) to prevent the formation of detrimental phases such as sigma, chi, carbides, nitrides, alfa prime, etc. To prevent the formation of detrimental phases, it is important to use rapid cooling methods and minimize the time spent at intermediate temperatures. The rate of cooling is critical; if cooling is too rapid, ferrite may predominate, whereas if cooling is too slow, austenite may be favored. Ultimately, this can result in an unbalanced duplex microstructure [2,3].

In the background of additive manufacturing, there is a growing interest also in DSS [5,6,7,8,9,10,11,12,13,14,15,16,17,18]. This is evidenced by the development of Super-DSS grade 2507 (EN 1.4410) powder, which is specifically designed for additive manufacturing processes. To achieve the desired mechanical and corrosion resistance properties of DSS, a balanced austenite–ferrite structure must be established. Fusion-based additive manufacturing processes, such as laser powder bed fusion (LPBF), undergo rapid heating, melting, solidification, and cooling due to their distinctive layer-by-layer deposition approach [12,19]. DSS compositions were originally designed for traditional manufacturing processes. However, the complex thermal cycles in AM make it difficult to achieve a well-balanced ferrite/austenite microstructure, which can affect the final properties [11]. The microstructures seen in AM DSS, in as-printed condition, are comparable to those found in the fusion zone microstructures of autogenously welded DSS [20]. The microstructures consist mostly of ferrite with small amounts of austenite found mainly at grain boundaries and within the ferritic matrix, as well as limited intragranular austenite. LPBF presents challenges in achieving a well-balanced duplex microstructure due to its rapid cooling rate, often resulting in a predominantly ferritic microstructure in the as-printed condition [5,8,21]. To overcome this limitation, post-processing annealing is necessary [13,16]. Additionally, stress-relief annealing can be applied after additive manufacturing to optimize the properties of DSS components. Recent research on additive manufacturing of DSS addresses such issues [9,10,13,15,16,21]. The printed microstructure of DSS can contain precipitates of secondary phases, such as sigma, and chromium nitrides, in addition to fully ferritic ones [13]. However, an appropriate solution annealing with a well-designed soaking time will result in a balanced ferrite-austenite microstructure with optimum fine grain size [13,16].

Stress relieving effectively minimizes residual stress and improves the material’s mechanical properties under as-built conditions. Stress relieving also results in dimensional stability and reduced risk of deformation or cracking during service of AM components. Stress relieving is generally not recommended for DSS as they are prone to the formation of detrimental and undesirable phases at the temperatures normally considered for stress relieving (i.e., 500 °C to 800 °C). Stress-relief annealing in most cases is performed in high temperatures, like quench annealing, depending on alloy composition, in the range 950 °C–1040 °C. In special cases, stress relieving may also be conducted at 500–550 °C [22]. Similarly, the maximum service temperature of all DSS grades is limited to below 315 °C according to ASME pressure vessel codes, and other codes specify even lower service temperatures, as low as 250 °C for SDSS. 

The properties of duplex stainless steels’ corrosion resistance are significantly influenced by the ratio between austenite and ferrite phases, as per the microstructural constituents [8,10,13,15,16]. Considering that heat treatment at an annealing temperature may lead to the precipitation of secondary phases, it is important to investigate its effect on the corrosion resistance of DSS manufactured through LPBF. The material properties should be compared with those of the as-built conditions. It was decided to investigate the effect of a stress-relieving heat treatment on the DSS microstructure, based on isothermal precipitation diagrams of wrought DSS alloys [2,4]. A significant research gap exists regarding the material properties and post-printing heat treatment processes for SDSS manufactured through LPBF. Recent studies have focused on the effect of LPBF processing conditions on DSS properties, but the specific properties of post-processed DSS, particularly after stress-relieving treatment, have not been adequately investigated. The need for more focused research around post-processing treatments is indicated by the lack of comprehensive research. 

In this paper, five states of printed DSS were compared: as-printed, after stress relieving (three types of temperature/time heat treatments), and after solution annealing. The research aimed to evaluate and compare the corrosion properties of LPBF SDSS in a 3.5% NaCl solution using the potentiodynamic anodic polarization technique and electrochemical impedance spectroscopy (EIS).

## 2. Materials and Methods

The chemical composition of the super duplex stainless steel (SDSS) powder, grade UNS S32750 (EN 1.4410)/Osprey^®^ 2507 VIGA made by Sandvik Osprey Ltd. is shown in Table 1. The 2507 powder is a gas-atomized powder with a spherical morphology of particle diameters ranging from 15 to 53 µm. It was used to print square samples (1.0 × 1.0 cm) using the laser beam-powder bed fusion (LPBF) method.

The laser beam-powder bed fusion (LPBF) printing process was performed on an AM125 RENISHAW printer (Renishaw, Wotton-under-Edge, UK) characterized by a ytterbium (Yb) fiber laser with a maximum laser power of 200 W, a scan speed of 2000 mm/s, and a wavelength of 1.074 nm. The components were manufactured on a mild steel platform under an atmosphere of the Ar inert gas at an oxygen level below 10 ppm. A meander scanning strategy was used, following a rotation of 67°, after every layer was laid. The printing parameters were optimized based on the lowest porosity in computer image analysis. The following printing parameters were used: laser power P = 180 W, hatch distance h = 120 µm, layer thickness t = 30 µm, and scan speed V = 300 mm/s. The energy density of the applied parameters was calculated according to the formula E_d_ = P/(V·h·t) J/mm^3^, and it was 166 J/mm^3^.

The super duplex stainless steel studied in this work was subjected to post-processing heat treatment, and the following five conditions were studied:

- As-printed, without any post-processing heat treatment (AS);
- Solution annealing of as-printed samples at 1100 °C for 15 min, fast cooling in water (SA);
- Stress relieving of as-printed samples at 300 °C for 5 h, slow cooling with the furnace (SR 300/5 h);
- Stress relieving of as-printed samples at 400 °C for 1 h, slow cooling with the furnace (SR 400/1 h);
- Stress relieving of as-printed samples at 550 °C for 5 h, slow cooling with the furnace (SR 550/5 h).

**Figure 2:**
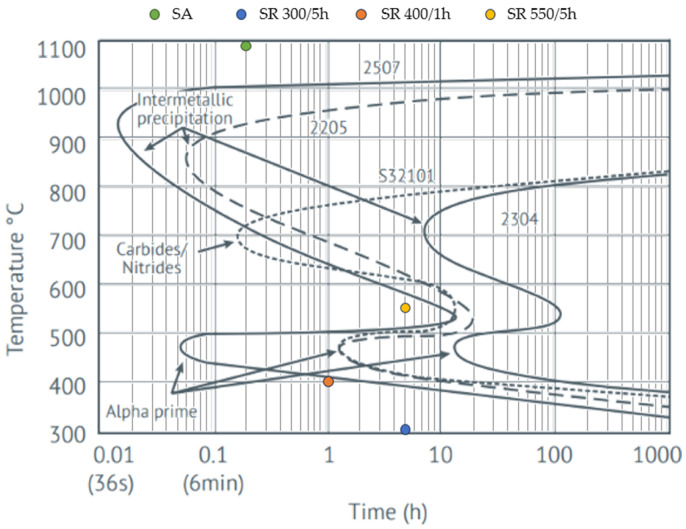
Heat treatment conditions on the isothermal precipitation diagram of DSS.

The tested LPBF states of the printed duplex steel are marked on Figure 2.

X-ray diffraction (XRD) patterns were collected using an X-Pert PRO instrument (Malvern PANalytical, Malvern, UK). For the X-ray diffraction analysis, a Co target, a scan rate of 0.01 step/s, and a scan range for 2θ between 30° and 120° were used. The X’Pert HighScore Plus was used for phase identification and quantitative analysis. The microstrain and dislocation density were calculated for the ferrite phase peaks. The full-width at half-maximum (FWHM, *β*) was estimated by a profile fitting. The crystallite size *D* (in units of nanometers) was then calculated with the Scherer equation (*D* = *kλ*/*β*cos*θ*), the dislocation density *δ* (nm^−2^) from *δ* = 1/*D*^2^, and the microstrain *ε* from *ε* = *β*/4tan*θ*. 

A LEICA MEF4A light optical microscope (LOM) (Leica Microsystems, Wetzlar, Germany) and a scanning electron microscope (SEM) Supra 35 from Zeiss Company (Jena, Germany) were used for metallographic examination. The metallographic specimens were fabricated using a conventional procedure consisting of grinding, emery paper polishing, and cloth polishing. Samples were electrolytically etched in 10% oxalic acid, and 3–6 volts were applied for 5–60 s.

The corrosion resistance was studied by potentiodynamic polarization test and electrochemical impedance spectroscopy (EIS). Corrosion tests were performed on an Atlas 0531EU & IA (Atlas-Sollich, Rebiechowo, Poland) potentiostat station in a 3.5% NaCl solution at room temperature, according to PN EN ISO 17475 standard [23]. The center of the sample’s cross-section was exposed to corrosion solution. Electrochemical tests were carried out in a three-electrode corrosion cell system with the test sample as the working electrode, an Ag/AgCl reference electrode (potential 207 mV at 25 °C), and a platinum wire as the auxiliary electrode. 

The open-circuit potential (E_OCP_) was measured for 1 h. Anodic polarization was recorded from E_ocp_−100 mV with step and potential rate changes of 1 mV/s until a current density of 1 mA/cm^2^ was reached. The polarity was then reversed, and the curve recorded to the initial potential. The Tafel extrapolation in AtlasLab software version 2.24 was performed and characteristic parameters related to electrochemical corrosion were measured: corrosion current density (J_cor_), corrosion potential (E_cor_), and polarization resistance (R_p_), which were determined according to the Stern–Geary Equation (1) [24], where βa and βc are the anodic and cathodic Tafel slope constant, respectively.

(1) $$R_{p}=\frac{\beta_{a}+\beta_{c}}{2.3\cdot I_{cor}\cdot (\beta_{a}+\beta_{c})}$$

The breakdown potential (E_br_) was determined as the point of depassivation and the inflection of the anode curve, and the repassivation potential (E_rp_) was determined as the point of intersection of the return and primary curves.

Corrosion behavior was also investigated by electrochemical impedance spectroscopy (EIS) analysis using the same test rig. EIS analysis was performed under equilibrium conditions at E_OCP_. The EIS frequency ranged from 10^4^ to 10^−3^ Hz and the voltage amplitude of the sinusoidal signal was 10 mV. The tests produced the impedance spectra of the circuit, which were presented as a Nyquist plot (a real (Z_r_)—imaginary (Z_i_) impedance curve) and the magnitude of the impedance (|Z|) as a function of frequency and the phase angle (θ) as a function of frequency. The impedance spectra were fitted to the electrical equivalent circuit (EEC) using AtlasLab and EC Lab software V11.41. Two tests per sample were performed in the EIS and potentiodynamic tests. The values obtained were very close to each other; thus, one representative result was selected and presented.

## 3. Results and Discussion

### 3.1. Microstructural Analysis

**Figure 3:**
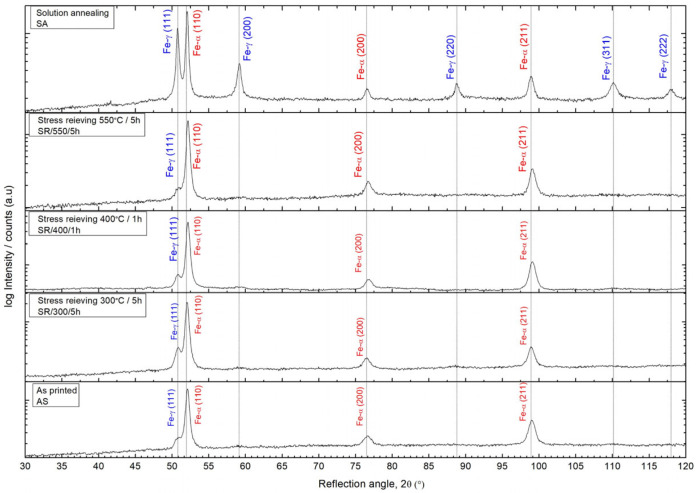
XRD patterns of LPBF-printed SDSS at as-printed, stress-relieved, and solution-annealed conditions.

LPBF-printed super duplex stainless steel (SDSS) reveals almost entirely ferritic microstructure in the as-printed state. The ferrite is the primary phase because of a high cooling rate of 10^6^–10^8^ °C/s in the LPBF process. The X-ray diffraction pattern (Figure 3) of as-printed SDSS, reveals strong ferrite phase peaks at Fe-α (110), (200), and (211), and one weak austenite phase peak at 2θ angle related to Fe-γ (111). The stress-relieving heat treatments at the studied temperatures do not affect the phase composition, but they do cause a minor increase in the intensity of the major austenite peak at Fe- γ (111). For the solution-annealed conditions, a fully balanced microstructure was formed, and both phases coexisted in a close-to-balanced share. In this case, peaks deriving from austenite, such as Fe-γ (111), (200), (220), (311), and (222), were clear. The austenite content is 5% in as-printed and stress-relieving conditions, and 52% for the solution annealing.

The full-width-half-maximum (FWHM, β) of the peaks corresponding to ferrite decreases with applied heat treatments, as shown in Table 2. The FWHM of the diffraction peaks can be related to various material properties, such as grain distortion, dislocation density, and residual stresses [25,26]. The widening of the X-ray peak and increase in FWHM are associated with an increase in stacking faults, structural disorder, and tensile stress in the material. Conversely, relaxation of stress decreases the FWHM. Additionally, the decrease in FWHM for the XRD peak may be related to a decrease in the hardness and density of point defects that alter crystallinity and grain boundary mobility [27]. Peak broadening in phases such as ferrite and martensite is often linked to a high number of lattice defects. Therefore, the peak shape can be used to predict the dislocation density in ferrite. Upon analysis of the XRD parameters of the ferrite peaks (Table 2), the number of lattice defects, calculated dislocation density (δ), and microstrains (ε) decrease with the application of heat treatment. A more significant effect is noticeable for higher stress-relieving temperatures. The crystallite size, D, increased after solution annealing, which is related to austenite formation and balanced duplex microstructure.

**Figure 4:**
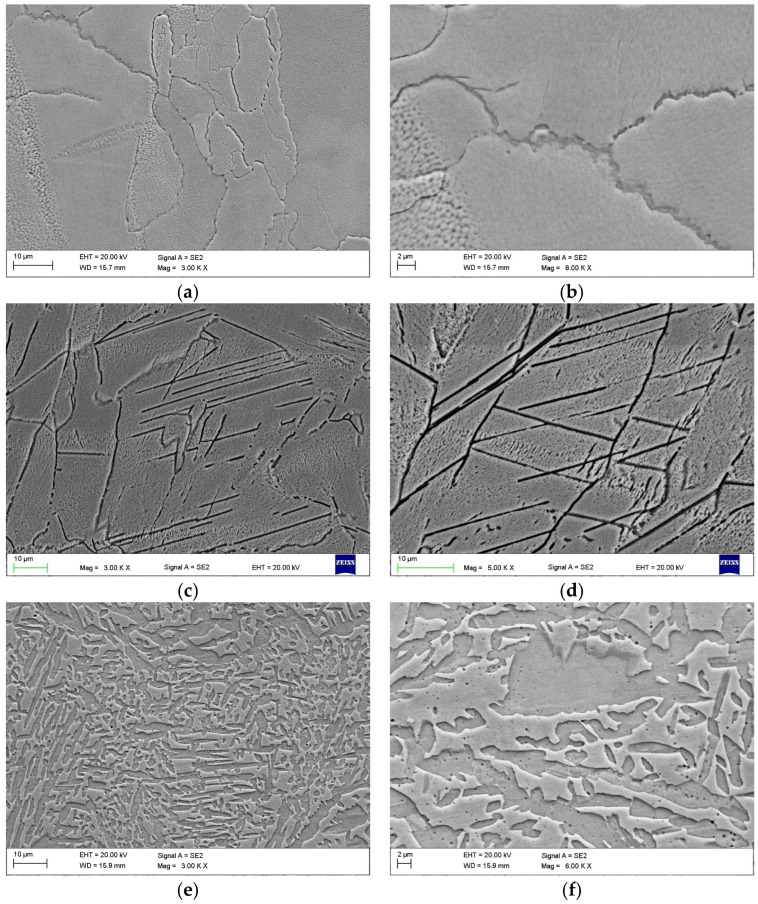
SEM microstructure of LPBF-printed SDSS (**a**,**b**) in as-printed conditions, (**c**,**d**) after stress relieving at 550 °C for 5 h, and (**e**,**f**) in solution-annealed conditions.

Figure 4 shows the microstructure of LPBF-printed SDSS. Under as-printed conditions, ferritic grains of varied shapes are visible (Figure 4a), with some weak austenite formed in the intergranular regions. The columnar ferrite grains are elongated in the build direction due to heat dissipation. At the boundary of the melting track, ferritic grains are inclined towards the center of the melt pool. In this region, numerous small round ferritic grains also occur. The grain size ranges from fine, round grains measuring 5–20 µm to elongated grains measuring 50–70 µm in length. Microcrystalline grain boundary austenite (GBA) was observed at the grain boundaries, exhibiting an allotriomorphic shape and morphology (Figure 4b). LPBF-related studies [5,6,7,8,9,11,12,14,21,28,29] have reported that extremely high cooling rates inhibit the formation of sigma phase in as-printed conditions. Additionally, an excess of nitrogen saturation within the ferrite matrix has been found to induce the formation of chromium nitride precipitates due to the formation of Cr-rich regions at grain boundaries (GB). Nonequilibrium segregation of alloying elements at GBs may also contribute to the precipitation of Ni–Mn–Si-rich phase (possibly the G phase) [8] close to nitride precipitations in as-printed DSS.

The microstructure of stress-relieved SDSS at 300 °C/5 h and 400 °C/1 h shows the same morphology as the as-printed one. Stress relieving at 550 °C for 5 h results in a different situation (Figure 4c,d). The ferritic matrix is saturated with nitrogen, and there is a low amount of austenite to dissolve nitrogen. As a result, CrN nitrides begin to precipitate. The driving force for nucleation, represented by free enthalpy, is higher for CrN than for Cr_2_N at temperatures below 1000 °C [30]. Under rapid cooling conditions that prevent the precipitation of austenite and Cr_2_N, the latter becomes more stable in equilibrium. In such situations, CrN precipitates before the more stable equilibrium phase Cr_2_N. The close similarity to the ferrite lattice and the lower activation energy for the nucleation of CrN are the reasons for this. The crystal structure of CrN nitride is primitive cubic, unlike the hexagonal Cr_2_N nitride. The low coherency of Cr_2_N nitrides with the ferrite and austenite lattices results in the precipitation of plate-shaped Cr_2_N nitrides on the ferritic–austenitic phase boundaries or low-angle grain boundaries in the ferrite. Additionally, Cr_2_N nitrides were detected within the ferrite matrix after long annealing times [30].

In the present study, chromium nitrides were manifested as intra-ferrite precipitates, on low-angle grain boundaries in the ferrite, forming long nitride bands that cut the ferrite grain from edge to edge (Figure 4c,d). The chromium nitride bands were oriented parallel to each other in the ferritic grains. This effect was only observed in stress-relieved SDSS at 550 °C/5 h. TEM studies will be performed to determine the type of nitrides formed in the SDSS steel after such heat treatment. The nitride precipitates in the stainless-steel structure after stress relieving at 550 °C/5 h will reduce the corrosion resistance, regardless of their crystalline structure [31].

The microstructure of SDSS was found to be balanced after the solution-annealing heat treatment, as shown in Figure 4e,f. In this condition, the microstructure is more fragmented, with fine austenite grains ranging from 5 to 20 µm in size in the ferrite matrix. The austenite was evenly distributed among the ferritic grains because of the formation of grain boundaries and intergranular austenite in the form of Widmanstatten laths within the ferritic grains. The SDSS produced by LPBF technology exhibits typical microstructural defects. Few small melting-related imperfections, such as gaps and lack of fusion near the melting track, were observed for the applied printing parameters. These imperfections can lead to the formation of cavities around partially melted powder particles and must be considered when analyzing corrosion resistance. In this study, all SDSSs were fabricated using the same LPBF process parameters. Subsequently, heat treatments were applied to one batch of samples.

### 3.2. Potentiodynamic Polarization Test

**Figure 5:**
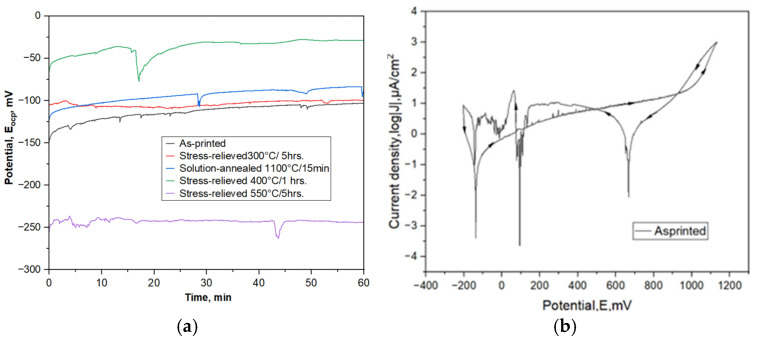

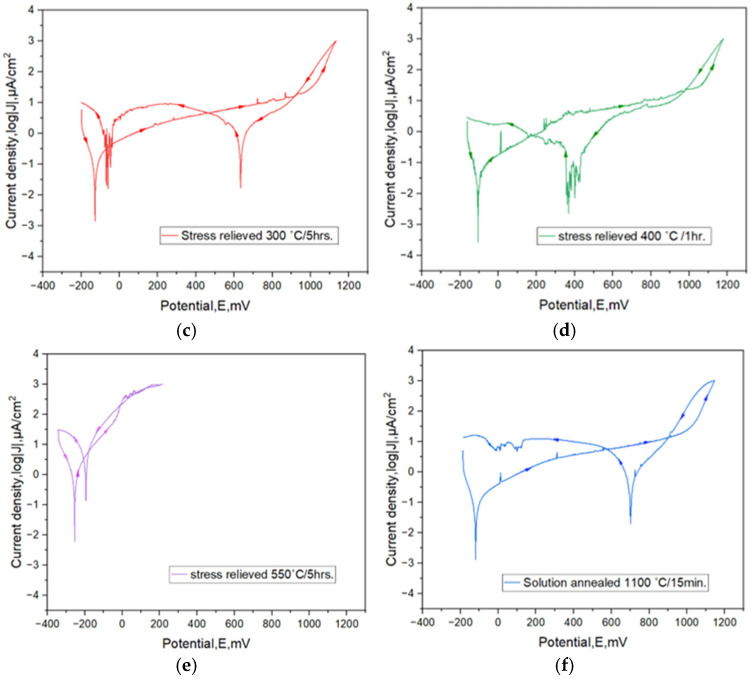
The potentiodynamic polarization-corrosion testing results of LPBF-printed SDSS: (**a**) open circuit potential; potentiodynamic polarization curves: (**b**) as-printed; (**c**) stress-relieved at 300 °C/5 h; (**d**) stress-relieved at 400 °C/1 h; (**e**) stress-relieved at 550 °C/5 h; (**f**) solution-annealed at 1100 °C/15 min. Arrows indicate the direction of the potential scan, the forward and backward scan.

The results of the potentiodynamic polarization testing are presented in Figure 5 and summarized in Table 3. The effect of heat treatment on open circuit potential (E_ocp_) values in stress-relieving and solution-annealed conditions was clearly noticeable (Figure 5a). For all SDSS conditions, a shift to positive E_ocp_ values after 60 min of immersion was observed (Figure 5a). The E_ocp_ of the SDSS tested ranged from −244 mV to −28.6 mV, with the stress-relieved sample at 400 °C/1 h having the higher value (−28.6 mV). The E_ocp_ of as-printed and stress-relieved samples at 300 °C/5 h show similar values, in the range −88 to −103 mV. The lowest shift toward negative values of E_ocp_ = −244 mV was observed for samples stress relieved at 550 °C/5 h. The lower open-circuit potential suggests faster material dissolution reactions and, as a result, a lower corrosion resistance.

Active, pseudo-passive, and transpassive states were identified in the SDSS anodic polarization curves (Figure 5b). The corrosion potential (E_corr_) of a material indicates its thermodynamic potential to either oxidize or passivate in a corrosive environment. When the corrosion potential is low, anodic reactions are more likely to occur. The corrosion processes start earlier on the as-printed SDSS, as evidenced by the lower value of E_corr_ compared to the other heat-treatment states. The amount of chloride ions (Cl^−^) present in the test solution is the factor that influences the detection of the passivation area. When testing in high-concentration solutions (Cl^−^), it is not uncommon for the passivation area and pitting potential to be barely visible on the cyclic potentiodynamic polarization curve. This behavior has also been reported for ferritic stainless steels (AISI 420 and 430) in solutions with chloride concentrations greater than 0.5 M [32,33]. The pseudo-passive range of super duplex stainless steel (SDSS) remains constant up to the breakdown potential (E_br_) under the conditions of this study. At the E_br_, the surface pitting is indicative of corrosion damage. The reverse polarization curve for all SDSSs shows a repassivation effect, and repassivation potentials were recorded (E_rp_), indicating conditions where actively corroding pits are repassivated and no further corrosion damage has occurred.

All the studied SDSS states undergo repassivation when reversing current polarization. The solution-annealed SDSS and stress-relieved SDSS at 400 °C/1 h also repassivated faster than other heat treatment conditions (Figure 5b). The repassivation potential (E_rp_) was remarkably consistent across all the SDSS states analyzed. The reverse loop produced in the diagram (Figure 5b) was extremely narrow. The E_br_ of the SDSS shows an average of 1090 mV, while E_rp_ = 923 mV, except for the stress relief at 550 °C/5 h, which shows a low breakdown potential and a short passive region. The passivity zone, indicated by a portion of the horizontal line after the corrosion potential peak in Figure 4b, can be identified from the characteristic potentials determined. For potentials above E_br_, pit formation can be observed, whereas for potentials above E_rp_, existing pits have undergone repassivation. New pits are certain to form when moving between E_br_ and E_rp_ potentials, but pre-existing pits on the surface may grow. The passive potential range (E_rp_–E_corr_) was close to 1200 mV, except for the stress-relieving heat treatment at 550 °C/5 h, where it is significantly reduced to only 391 mV. The E_sec_ potential, which refers to the anodic–cathodic transition on the reverse anodic polarization curves after reaching the breakdown potential, follows the same trend.

The studied heat treatment conditions greatly influence the corrosion resistance of LPBF-printed SDSSs (Table 3). As expected, solution annealing improves corrosion resistance; the polarization resistance (R_pol_) increased from 126 kΩ·cm^2^ to 149 kΩ·cm^2^ compared to the as-printed conditions. Comparing stress-relieving heat treatments, stress relieving at 300 °C/5 h has no effect on polarization resistance, while in case of 400 °C/1 h a double increase in R_pol_ to 301 kΩ·cm^2^ may be seen, meaning significant improvement of corrosion resistance. The opposite situation took place after stress relieving at 550 °C/5 h, where polarization resistance remarkably decreased to 17.68 kΩ·cm^2^.

It is known that the two-phase structure of duplex stainless steel shows better corrosion resistance when the proportions of the two phases are close to each other. Too low or too high a proportion of one phase (exceeding the recommended limit of 30/70) increases the corrosion rate of wrought DSS. This is well known from the welding practice of DSS [4,34]. In the case of the LPBF-printed SDSS studied, similar electrochemical parameters (R_p_, R_rp_) were obtained as in the solution-annealed state, which should theoretically improve corrosion resistance more effectively. Although the as-printed SDSS has a predominantly ferritic structure with up to 5% austenite, after solution annealing it contains approximately 52% austenite. On the other hand, the use of stress-relief annealing, which is mainly aimed at eliminating the effects of significant structural stresses after printing, provided the opportunity to further improve the corrosion resistance of the SDSS. Certainly, heat treatment at 400 °C/1 h gives a significant increase in corrosion resistance compared to the material in the as-printed condition, and even better corrosion resistance than after solution annealing, in studied cases. In this context, if the main requirement of the material in an application is high corrosion resistance, short annealing at 400 °C/1 h will certainly improve the LPBF corrosion resistance of the printed SDSS steel.

The 5 h stress-relieving treatment at 550 °C significantly decreased the corrosion resistance, as evaluated by comparing the polarization resistance values. Prolonged heating at such conditions caused precipitation of chromium nitrides, which may be linked to the impairment of corrosion resistance.

### 3.3. Electrochemical Impedance Spectroscopy

**Figure 6:**
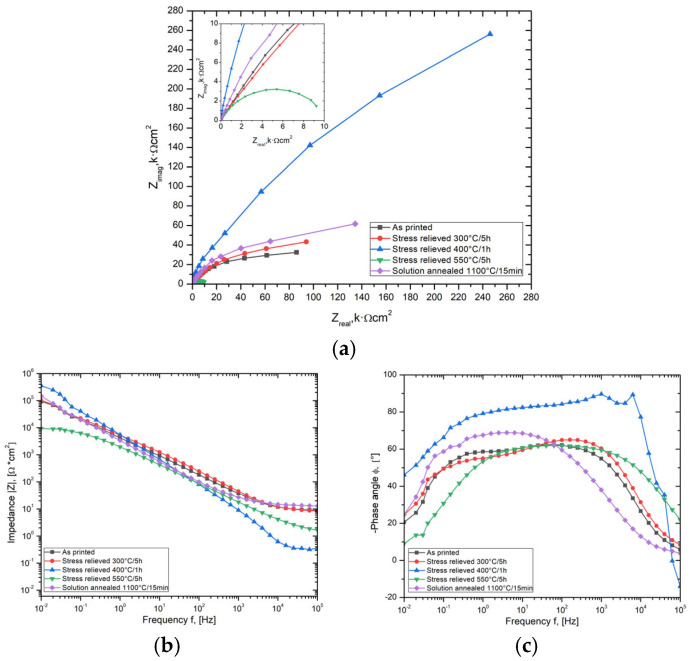
Impedance spectra of LPBF-printed SDSS for as-printed, stress-relieved, and solution-annealed conditions: (**a**) Nyquist plot; (**b**) impedance modulus as a function of frequency; (**c**) phase angle as a function of frequency.

Electrochemical impedance spectroscopy (EIS) measurements were conducted in a 3.5% NaCl solution to evaluate the electrical properties and compare the results with an analysis of the anodic polarization curve (Figure 6). The Nyquist plot (Figure 6a) shows the typical impedance curve of thin oxide films arranged in semicircles. The waveforms of all impedance curves are close to each other, apart from the state after stress-relieving heat treatment at 550 °C/5 h, where the impedance curve reaches several times lower values. The impedance spectra show the highest values for samples stress relieved at 400 °C/1 h, then for solution-annealed, and the lowest course of the impedance curves is for samples stress relieved at 550 °C/5 h. The variations in the impedance modulus of the corrosion system are interpreted to mean that higher values are associated with improved surface barrier properties and, hence, better corrosion resistance. Thus, the greater the radius of curvature in the Nyquist plots, the better the corrosion resistance of the sample surface.

The Bode diagrams presented in Figure 6b,c illustrate the relationship between the impedance modulus and the phase shift angle (ϕ) in function of logarithmic frequency. These diagrams provide a means for evaluating the efficiency of Cl^−^ ions in transmitting across joint surfaces and their contribution to corrosion degradation. The shift angle, which represents the delay in the system’s response to the voltage signal, directly affects the impedance value. The impedance can be represented as pure resistance when the current and voltage are in phase. When the phase angle (ϕ) approaches −90^°^, the impedance can be characterized as a capacitor. Conversely, when the phase angle approaches +90^°^, it can be defined as an inductor. The trajectory of the phase shift angle curves and their magnitude enables the evaluation of the effectiveness of the protective barrier in preventing corrosion damage on the surface of the tested material. Assuming the shortest phase shift angle implies that the barrier is the thickest and most dense [35].

The impedance modulus (|Z|) as a function of log. frequency (Figure 6b) follows a similar course for the as-printed conditions after heat treating at 300 °C/5 h and after solution annealing. However, when heat treated at 400 °C/1 h and 550 °C/5 h, the modulus value gradually decreases at high frequencies, particularly for the 500 °C/5 h condition. A high modulus value over a wide frequency range indicates higher corrosion resistance.

The phase shift angle (ϕ) is a function of the log. frequency (Figure 6c) shows a similar pattern for the as-printed and heat-treated conditions at 300 °C/5 h. The maximum phase shift angle occurs over a wide frequency range from 10^−1^ Hz to 10^3^ Hz. For the as-printed condition, the phase shift angle has a similar shape but higher values at lower frequencies. However, when heat treated at 400 °C/1 h, there is a marked increase in the phase shift angle and a plateau from a value of 10^−1^ Hz to a maximum at 10^3.5^ Hz. The phase shift angle for the 550 °C/5 h heat treatment is like the curve for the 400 °C/1 h condition, but one can clearly see lower angle values at lower frequencies with the same maximum of around 10^3^.^5^ Hz. In this case, the phase angle is high for high frequencies, whereas for the 400 °C/1 h condition it drops to negative values.

**Figure 7:**
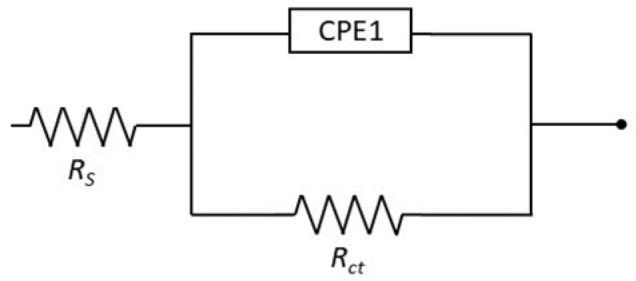
Equivalent electric circuit (EEC) for LPBF-printed SDSS studied in NaCl solution.

The characterization of the interface impedance of the tested samples was carried out by approximating the experimental EIS data with an electrical model of the equivalent circuit, as shown in Figure 7. A single constant equivalent circuit was used for all samples, which can be interpreted as a single oxide layer on the surface. The electrical equivalent circuit (EEC) adopted was composed of two resistors and a constant-phase element (CPE). The R_s_ value corresponds to solution resistance and R_ct_ is the charge transfer resistance in the phase interface that may be directly linked to the corrosion resistance of the surface. Because of the dispersion of time constant and dispersion due to surface adsorption/diffusion processes, known as the kinetic dispersion effect [36], constant phase element (CPE_1_) was selected to describe capacitance in the EEC. The CPE_1_ is described by capacitance parameter Y_dl_ and n that is CPE exponent and its impedance is defined as Z_CPE_ = 1/Y_dl_ (jω)^n^. The double layer capacitance C_dl_ of the system was then computed with the “Pseudocapacitance” tool in EC-Lab software [37]. Table 4 summarizes the calculated EIS parameters.

A comparison of the corrosion resistance of LPBF-printed SDSS is made by comparing the resistance R_ct_, which is related to the charge transfer (or polarization resistance). Like the Tafel analysis of the anodic polarization curves, the material in as-printed conditions and after solution annealing at 300 °C/5 h shows similar values of R_ct_ resistance. For solution-annealed conditions, there was an increase in R_ct_ resistance of approximately 20%, confirming the positive effect of such heat treatment. The greatest differences were found for the 400 °C/1 h heat treatment, which causes a doubling of the R_ct_ resistance value, i.e., a significant improvement in the corrosion resistance of the material. On the other hand, for the heat treatment at 550 °C/5 h, there was a reduction in the charge transfer resistance to 10% of the initial values specific to the as-printed condition without heat treatment.

The double-layer capacitance was found to be inversely proportional to the thickness of the oxide layer formed on the surface [38,39]. The capacitance and the associated thickness of the oxide layer depend on the heat treatment applied. A decrease in the double-layer capacitance C_dl_, indicating an increase in the thickness or dielectric strength of the passive layer, reflects an improvement in corrosion resistance. This effect is more pronounced with stress relieving at 300 °C/5 h, where the C_dl_ is low, so a thicker oxide layer can be expected to be responsible for the improvement of corrosion. A long heat treatment time (i.e., 5 h) favors the growth of the oxide layer, whereas a short heat treatment (1 h) results in a thinner passive layer and a higher C_dl_ capacitance value. The highest double-layer capacitance (C_dl_), i.e., a thinner passive layer, was found for stress-relieving heat treatment at 400 °C/1 h. Nevertheless, the corrosion resistance of this treatment condition was the best of all the conditions analyzed. It follows that temperature is a more important parameter for the effective improvement of corrosion resistance than the duration of stress relieving.

The n exponent of the CPE element ranged from 0.7 to 0.77 for most of the stated conditions, except for the stress relieving at 400 °C/1 h, where it was 0.85, presenting values closer to the capacitive properties (when n = 1, the CPE represents pure capacitance). The variation of the system’s behavior from the ideal is indicated by the deviation. It causes a deviation from ideal capacitive behavior. This deviation is present in all samples due to surface heterogeneities at both the micrometric and atomic scale. These heterogeneities include roughness, a polycrystalline microstructure, surface disorder in the form of dislocations and steps, chemical inhomogeneities, and adsorption phenomena. The solution had the smallest resistance among the analyzed results in these conditions. Lower solution resistance is generally preferred for accurate EIS measurements. This is because it minimizes potential drops that are not related to the electrochemical reactions of interest, allowing for a clearer distinction of the processes occurring at the electrode–electrolyte interface. From the above discussion, it can be concluded that the most favorable electrochemical parameters from the EIS test were obtained from the samples heat treated at 400 °C for 1 h.

The nonuniform distribution of residual stresses can cause variable corrosion rates across the material’s surface. Stress-relieving heat treatment can help achieve a more uniform stress distribution, which may contribute to improved and consistent corrosion resistance. Residual stress can trigger and develop localized corrosion, such as pitting and crevice corrosion.

## 4. Conclusions

Based on the above results, it can be concluded that a suitably selected stress-relieving time and temperature can improve the corrosion resistance of LPBF-printed SDSS steel.

LPBF-printed SDSS in as-printed conditions exhibits a primarily ferritic microstructure that can be easily balanced by solution-annealing heat treatment with slight improvement of corrosion resistance. SDSS in as-printed conditions shows varied ferritic grain shapes, with some austenite formation in intergranular regions and allotriomorphic microcrystalline grain boundary austenite. The solution annealing causes balanced and more fragmented microstructures.

Potentiodynamic polarization tests and electrochemical impedance spectroscopy (EIS) results indicated that stress relieving at 300 °C for 5 h and stress relieving at 400 °C for 1 h improved corrosion resistance, as evidenced by increased polarization resistance and more favorable EIS parameters. In contrast, stress relieving at 550 °C for 5 h significantly decreased corrosion resistance due to chromium nitride precipitation. Interestingly, stress relieving at 400 °C for 1 h significantly improved corrosion resistance, potentially offering a practical approach to enhancing the LPBF-printed SDSS’s performance in corrosive environments.

Further studies will explore the optimization of heat treatment parameters and their effect on the mechanical properties of LPBF-printed SDSS (i.e., shorter time at 550 °C and longer time at 400 °C), as well as investigate alternative stress-relieving temperatures to improve corrosion resistance and mechanical performance.

## Figures and Tables

**Table 1:** Chemical composition of 2507 super duplex stainless steel powder.

Element	Fe	Cr	Ni	Mo	Mn	Si	Cu	S	P	C	N	O
wt. %	65.01	25.2	6.3	3.8	0.4	0.3	0.15	0.001	0.024	0.0160	0.35	0.0170

**Table 2:** XRD diffraction pattern parameters.

Condition	Parameter	Fe-α’ (110)	Fe-α’ (200)	Fe-α’ (200)	*β*, 2Θ° (med.)	*D* (nm)	*δ* × 10^−3^ (nm^−2^)	*ε* × 10^−3^
SA 1100 °C/15 min	2Θ°	52.05109	76.58382	98.92256	0.631	20.44	2.78	3.46
	*β*, Θ°	0.37954	0.75392	0.75971				
SR 550 °C/5 h	2Θ°	52.16993	76.75633	99.11614	0.762	14.47	5.36	4.31
	*β*, Θ°	0.45318	1.01012	0.82259				
SR 400 °C/1 h	2Θ°	52.16231	76.81057	99.11002	0.825	13.47	6.14	4.62
	*β*, Θ°	0.51106	1.07759	0.8878				
SR 300 °C/5 h	2Θ°	52.04914	76.51321	98.94692	0.854	13.06	7.07	4.94
	*β*, Θ°	0.49392	1.19726	0.87041				
AS	2Θ°	52.10395	76.64378	99.02586	0.903	12.16	7.85	5.21
	*β*, Θ°	0.50937	1.24371	0.95526				

AS—as-printed condition; SR—stress-relieving condition; SA—solution-annealing condition.

**Table 3:** Results of the potentiodynamic (Tafel analysis) test on the stress-relieved, solution-annealed, and as-printed AISI SAF 2507 DSS.

Sample Designation	E_OCP_	E_br_	E_rp_	E_rp_–E_corr_	E_sec_	J_corr_	β_a_	β_c_	E_corr_	R_p_
	mV	mV	mV	mV	mV	µA/cm^2^	mV	mV	mV	kΩ·cm^2^
AS	−103	999	1084	1231	649	140.63	195.73	51.98	−147.17	126.82
SR 300 °C/ 5 h	−99.7	996	1083	1225	634	166.60	255.88	59.11	−142.86	125.14
SR 400 °C/1 h	−28.6	1055	1127	1210	368	59.06	225.50	50.05	−83.81	301.10
SR 550 °C/5 h	−244	−96.3	130	391	−195	1159.0	117.99	78.67	−261.03	17.68
SA 1100 °C/15 min	−88.2	1011	1102	1238	701	131.89	295.36	53.86	−136.35	149.35

AS—as-printed condition; SR—stress-relieving condition; SA—solution-annealing condition.

**Table 4:** EIS parameters of LPBF-printed SDSS for as-printing, stress-relieved, and solution-annealed conditions.

Sample Designation	R_S_	Y_dl_ µF·s^(n−1)^·cm^−2^	n	C_dl_	R_ct_
	Ω·cm^2^			µF·cm^−2^	kΩ·cm^2^
AS	7.980	83.61	0.70	9.691	78.34
SR 300 °C/ 5 h	6.950	49.57	0.70	4.983	94.80
SR 400 °C/1 h	0.370	51.24	0.85	23.59	240.65
SR 550 °C/5 h	1.210	126.40	0.77	16.83	9.260
SA 1100 °C/15 min	13.55	70.25	0.77	16.93	121.5

AS—as-printed condition; SR—stress-relieving condition; SA—solution-annealing condition.

## Data Availability

The data are included in the article and are available on request from the corresponding author.
